# Detecting Falls as Novelties in Acceleration Patterns Acquired with Smartphones

**DOI:** 10.1371/journal.pone.0094811

**Published:** 2014-04-15

**Authors:** Carlos Medrano, Raul Igual, Inmaculada Plaza, Manuel Castro

**Affiliations:** 1 Computer Vision Lab, Aragon Institute for Engineering Research, Zaragoza, Spain; 2 EduQTech Group, Escuela Universitaria Politecnica, University of Zaragoza, Teruel, Spain; 3 Electrical and Computer Engineering Department (DIEEC), Spanish University for Distance Education (UNED), Madrid, Spain; University Hospitals of Geneva, Switzerland

## Abstract

Despite being a major public health problem, falls in the elderly cannot be detected efficiently yet. Many studies have used acceleration as the main input to discriminate between falls and activities of daily living (ADL). In recent years, there has been an increasing interest in using smartphones for fall detection. The most promising results have been obtained by supervised Machine Learning algorithms. However, a drawback of these approaches is that they rely on falls simulated by young or mature people, which might not represent every possible fall situation and might be different from older people's falls. Thus, we propose to tackle the problem of fall detection by applying a kind of novelty detection methods which rely only on true ADL. In this way, a fall is any abnormal movement with respect to ADL. A system based on these methods could easily adapt itself to new situations since new ADL could be recorded continuously and the system could be re-trained on the fly. The goal of this work is to explore the use of such novelty detectors by selecting one of them and by comparing it with a state-of-the-art traditional supervised method under different conditions. The data sets we have collected were recorded with smartphones. Ten volunteers simulated eight type of falls, whereas ADL were recorded while they carried the phone in their real life. Even though we have not collected data from the elderly, the data sets were suitable to check the adaptability of novelty detectors. They have been made publicly available to improve the reproducibility of our results. We have studied several novelty detection methods, selecting the nearest neighbour-based technique (NN) as the most suitable. Then, we have compared NN with the Support Vector Machine (SVM). In most situations a generic SVM outperformed an adapted NN.

## Introduction

Falls in the elderly are one of the major health risks that affect their quality of life, threatening their independent living. According to the World Health Organization [1] approximately 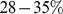 of people aged 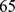 and over fall every year, increasing to 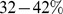 for those over 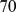 years of age. They typically suffer moderate to severe injuries such as bruises, hip fractures or head trauma. Therefore, fall prediction, prevention and protection are major areas for current research. In recent years, the number of proposed fall detection systems and algorithms developed has increased dramatically. An overview of the topic can be found in [2], [3] and in our recent review [4], where we highlight the current challenges and trends in this field. Fall detection systems can be based on sensors deployed at home (cameras, pressure or motion sensors) or on accelerometers carried by the user. Every technique has its merits and demerits. Accelerometer-based systems are very popular since they measure the body's movement directly. Devices specially made for fall detection are worn attached to the body [5]–[10], but there is a new trend towards using the accelerometers integrated into smartphones [11]–[16], which is the solution we have adopted in this work. Smartphones are powerful devices and applications targeting older people are often related to topics such as health, wellness, safety and mobility [17]. Integration of many functionalities into a single device is very attractive. Usability might still be a concern but we think that in the near future more and more people will get used to these devices and that a careful design can help to overcome technology barriers [16], [18].

Regarding fall detection methods, those based on thresholding are typical when using acceleration readings. For instance, the peak value is supposed to exceed a given threshold during a fall [6], [8], [11], [13]. Other approaches use more sophisticated Machine Learning methods [10], [14]. Threshold-based methods can be implemented with little effort and their computational load is minimal, but they are limited when facing real situations. The Machine Learning approach is more sophisticated and leads to better detection rates. Nevertheless, these techniques use more computation resources and can be difficult to implement. At the moment there is no widely accepted method among researchers in this field. Besides, some impediments prevent a fair comparison of methods. There is no public data base available and each author decides which kind of ADL and falls are included in the study and how they are recorded. Many details in this process will have an impact on the classifier's performance. In this regard, there is an European project aiming to provide solutions for health promotion and fall prevention [19]. An android app has been developed to acquire inertial sensors data with the hope of recording real-world falls in a standardized format [19], [20].

Although some functional prototypes have been implemented and several commercial products are available on the market, the fact is that there is a rejection of such systems by both the wearer and the caregiver due to the rate of false alarms, which results in inappropriate alerts, among some other issues [2]. Bagala et al. [21] performed an extensive comparison of the most popular acceleration-based fall detection techniques. They found that the number of false positives per day in real scenarios ranged from 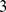 to 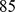 depending on the specific technique, showing a decrease in performance with respect to laboratory environments. This number is still not acceptable, which leads to device rejection. Therefore, to improve the level of penetration of these systems it is essential to find a robust fall detection method.

The existing fall detection studies use traditional supervised techniques, which need labelled samples for both activities of daily life (ADL) and falls. Falls have traditionally been simulated by young volunteers. This may explain the loss in performance when used in real situations, since a system trained with data from young subjects is then carried by old people. Although collecting data from real falls of old people is extremely difficult, data from ADL can be recorded while they carry smartphones in their everyday life, thus registering a large number of true movements. Taking this into account, the aim of the present study is to explore the use of techniques based on real data which could adapt to different conditions. Therefore we have resorted to some novelty (or anomaly) detection methods [22] which need only labels from one class, the “normal” one. Similar approaches were also proposed in [23] but using acoustic signals and not acceleration readings as we do in this paper. These methods only use ADL to train the detector. Once trained, a new input is considered as a novelty, a fall in our case, if it is very different from the ADL training data. Several techniques differ in the way they measure this difference.

An adaptive smartphone application based on novelty detection should include two functions. The classifier itself and the learning procedure in which new ADL are collected and the classifier is re-trained as often as possible. Initially, the application would be based on pre-defined typical ADL but after some time the classifier would be completely adapted to the user's movements, smartphone's characteristics, phone position, etc. Besides, if something changed, for instance there were some drift in user's mobility, the detector would adapt again automatically since the learning process never stops. Our first guess was that such a system had several advantages that cannot be found in traditional supervised techniques:

- It could be easily personalized when the detector is carried by a new user, without the need to ask him or her to simulate falls.
- Its behaviour could be easily adapted to new conditions: changes in user's movements, position of the phone, phone use, accelerometer characteristics, etc.
- It would not rely on fall simulations, which may not represent every possible fall situation. It is unclear how a supervised method would react when tested with a kind of fall that was not in the training set.

Thus, the overall goal of this paper is to investigate the use of novelty detection techniques for fall detection. Although we have not collected real fall data from older people, we want to test the adaptability of novelty detectors to different conditions as well as to compare a state-of-the-art generic supervised method with an adapted novelty detector. For that purpose, the generic method will face situations for which it was not trained, while the novelty detector will use the right ADL in each case. Our off-line analysis has considered the following items: the person who carries the detector, the kind of fall, the characteristics of the smartphone and where it is placed. In this paper, we will show under which circumstances an adapted classifier is a better option.

In addition, the data sets that we have generated for this work can be freely downloaded (see next section). Up to our knowledge, this is the first time such information has been made publicly available. We hope this improves the reproducibility of our results and helps other researches to compare their methods.

## Materials and Methods

### Subjects

The first step of the study was to identify typical fall and ADL patterns. The study involved young and middle-aged volunteers, since it would be inappropriate to subject elderly people to simulate falls. Ten participants, 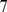 males and 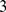 females, were recruited for this study. Volunteers ranged from 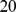 to 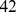 years old (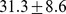 years), body mass 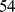 to 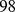 kg (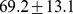 kg) and height from 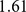 to 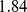 m (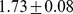 m).

### Ethics Statement

The study protocol was approved by the Ethical Committee for Clinical Research of Aragon (CEICA). All subjects received oral and written information about the study, and written informed consent was obtained from them.

### Falls and ADL

Participants performed eight different types of simulated falls: forward falls, backward falls, left and right-lateral falls, syncope, sitting on empty chair, falls using compensation strategies to prevent the impact and falls with contact to an obstacle before hitting the ground. The first six fall types were selected following the proposal of Noury et al. [24] for the evaluation of fall detectors and previous research that showed these are the most common types of falls among the elderly people [6], [8], [25]. The last two types were included as they are common in real-life scenarios according to the studies of Kangas et al. [26] and Klenk et al. [27]. Each fall was repeated three times for a total of 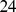 fall simulations per subject. Falls were completed on a soft mattress in a laboratory environment. During the falls participants wore a smartphone in both their two pockets (left and right). Thus a total of 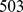 records were obtained (due to some technical problems some falls had to be repeated in a few cases, so this number is higher than 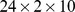). The ADL study was carried out under real-life conditions. Participants carried a smartphone in their pocket for at least one week to record everyday behaviour. On average, about 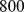 ADL records were collected from each subject during this period. An additional ADL data set was obtained by 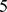 of the volunteers who carried the phone in a hand bag for about one week, obtaining roughly 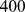 ADL per subject. During fall simulations volunteers also carried two hand bags with smartphones, allowing us to obtain another fall data set with 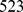 records. These additional data sets were only used to study the influence of the phone's position.

After a pre-processing step (see below), each ADL or fall was transformed into a vector, which we call a record in this paper, composed of 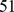 acceleration values taken at 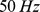 and centred at the peak.

### Data acquisition and processing

During the monitoring period, acceleration signals from the built-in triaxial accelerometer of a smartphone were acquired continuously. The devices were Samsung Galaxy Mini phones running the Android operating system version 2.2. The sampling rate was not stable, with a value of about 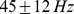. We stored in an internal file acceleration signals from the three axes together with temporal information. During the daily life monitoring, whenever a peak in the acceleration magnitude was detected to be higher than 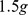 (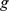 = gravity acceleration), a new entry was appended to the file. This value is below the acceleration peak during falls reported by previous authors [6], [11], [13]. Each entry included information in a time window of 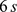 around the peak. During each fall simulation, we also got a 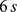 width time window around the highest peak. Then, the file was transmitted wirelessly to a server once the monitoring period was over. Afterwards, the offset error of each axis was removed, the acceleration magnitude was calculated and an interpolation was performed to get a sample every 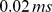 (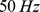). For the analysis presented in this paper, we kept only the central 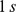. In this way, each ADL or fall was transformed into a vector with 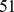 values.

The data sets together some Python scripts to handle them are available for download at the following address: http://eduqtech.unizar.es/en/fall-adl-data/.

### Algorithms and their evaluation

We have used several novelty detection techniques, see [22] for a brief explanation and further references. All the selected methods can be trained only with ADL. Despite its simplicity, k-nearest neighbour (kNN) has shown good performance in many practical applications. It needs an initial set of ADL training records. Given a new record, the novelty score is the distance to its k-nearest neighbour in the training set. If the novelty score is higher than a given threshold, the new record is classified as a novelty, a fall in our case. By varying the threshold, the receiver operating characteristic curve (ROC) can be depicted. In this paper, the area under the ROC curve (AUC) has been selected as the main figure of merit of the classifiers. To compare with previous studies, a specific value of sensitivity (SE) and specificity (SP) is also provided. These values have been obtained by selecting the point that maximized their geometric mean, 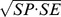, in a ROC curve averaged over the cross-validation results. These figures of merit are insensitive to differences in size between ADL and fall data sets.

Two variants of kNN have also been considered. In the first case, the novelty score was also obtained as the sum of distances to the k nearest neighbours (kNN-sum). Another group of tests was done using a K-means clustering before applying a 1NN rule (K-means+NN). In this way, the number of records was reduced and only the cluster centres were considered afterwards.

Finally, we have also tested a more sophisticated novelty detection algorithm, the One-Class Support Vector Machine (One-Class SVM), which tries to estimate the support of a probability distribution, thus allowing to reject samples that are unlikely to have being obtained from that distribution [28]. In the raw One-Class SVM, the sign of the distance to the hyperplane found in the training process determines the class. By thresholding this distance, the ROC curve can be drawn.

With respect to traditional supervised methods, we have selected one of the state-of-the-art classifiers, SVM with a radial basis function as kernel, which have been successfully applied for non-linear problems in high dimension spaces. As in the previous case, thresholding the distance to the hyperplane allows depicting its ROC curve.

All the algorithms were implemented in Python. For K-means, One-Class SVM and SVM we used the scikit-learn package (version 10.0, which comes as a package for Ubuntu 12.04) [28]. The SVM Python interface links in turn to libSVM [29]. One important point in our case was the difference in size between the ADL and fall data sets. To account for it, the library allows defining a weight parameter for each class to deal with unbalanced data sets. In this way, the error penalty is multiplied by the corresponding weight when training SVM. Thus, the fall weight was set to the number of ADL and vice versa. Parameters in SVM and One-Class SVM (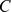, 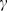 or 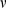) were selected using a grid search to look for the values that minimized a weighted error in an internal cross-validation, that is, a cross-validation using only the training set. The weighted error accounted again for different sizes in the ADL and fall data sets. The kNN and kNN-sum novelty detectors were implemented with our own code. We selected the Euclidean distance as the distance measure.

The evaluation of the algorithms was different depending on the goal. First, we selected the most suitable detector among the novelty detection algorithms. For the comparison between them, we used a 10-fold cross-validation, dividing the ADL data set into 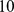 groups. Falls were needed mainly to test the algorithms. The only particular aspect was that we had to kept a 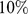 of falls out of the testing phase in each run. This group and the ADL training set were used to select the parameters of the One-Class SVM classifier.

After selecting the best novelty detector, we compared it with SVM. We have summarized in figure 4 the conditions applied to perform this comparison. First, we performed a standard 10-fold cross-validation. Then, we simulated situations in which a generic SVM faced conditions that were not present in its training set and compared its performance with that of an adapted novelty detector. This was achieved by selecting suitable train and validation sets for cross-validation:

- To study the effect of new types of falls, the cross-validation was carried out by fall type. In this case, in each run of the cross-validation all the falls of a given type were kept for validation and the remaining falls were used for training.
- The sampling rate is another source of variation because not all the smartphones sample at the same frequency. Let's assume that a generic SVM has been trained with records at a given sampling frequency and over a given time span, in our case 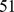 samples at 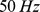, a bit more than 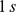. Thus, the classifier requires a 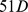 vector as input. If the classifier were run in a new phone sampling at a lower frequency, the data acquired by the phone would have to be interpolated. To simulate such a situation, the validation set for SVM was subjected to a two-step process. Firstly, the original data set was subsampled at a lower frequency. In this way we obtained the kind of records that would be acquired in the new phone. Secondly, since SVM still requires a 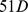 vector at 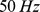, the records obtained after the first step were interpolated again to 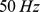 and the corresponding samples around the peak were selected to feed SVM. In this two-step process, the original data were not recovered exactly and some information was lost. On the other hand, an adapted novelty detector would use directly the data acquired with the new phone, including those to train the detector itself. Thus, both training and validation sets for the novelty detector were obtained by subsampling the original data.
- The position of the phone has also an impact on the detector's performance. A 10-fold cross-validation was performed for the novelty detector using only data acquired with the smartphone in a hand-bag. In each run, SVM was tested against the same validation set, but trained using different ADL and falls each time selected from those obtained with the phone in the pocket. For a fair comparison, the sizes of ADL training sets for the novelty detector and SVM were the same. In this way, we simulated again an adapted system based on a novelty detector, in which new records for training can be acquired by each user with the phone in a different position.
- Finally, we studied the effect of personalization. For each subject, we tested the algorithms with part of his or her own data. The novelty detector was trained with the rest of his or her own data while SVM was trained with data from the remaining people. Thus, the novelty detector was personalized whereas SVM was generic. For a fair comparison, we took the same number of ADL records in both training sets. This was repeated ten times for cross-validation.

An implicit assumption of our proposal is that personalization should improve the detector's performance. To check this issue, we have also compared the novelty detector with and without personalization. This comparison followed the same selection of train and validation sets as explained in the last paragraph (in fact, we took advantage of the same cross-validation runs to estimate the performance and we will present the results in the same table).

## Results

**Figure 1:**
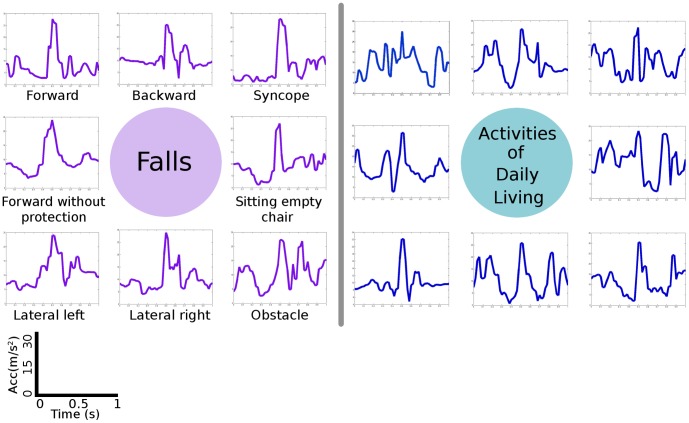
Some examples of acceleration shapes obtained during falls and ADL.

Two data sets, falls and ADL, were collected from ten volunteers. Some examples of falls and ADL acceleration shapes are shown in figure 1. These data sets were used for the off-line analysis that follows.

### Comparing and selecting a novelty detector

**Figure 2:**
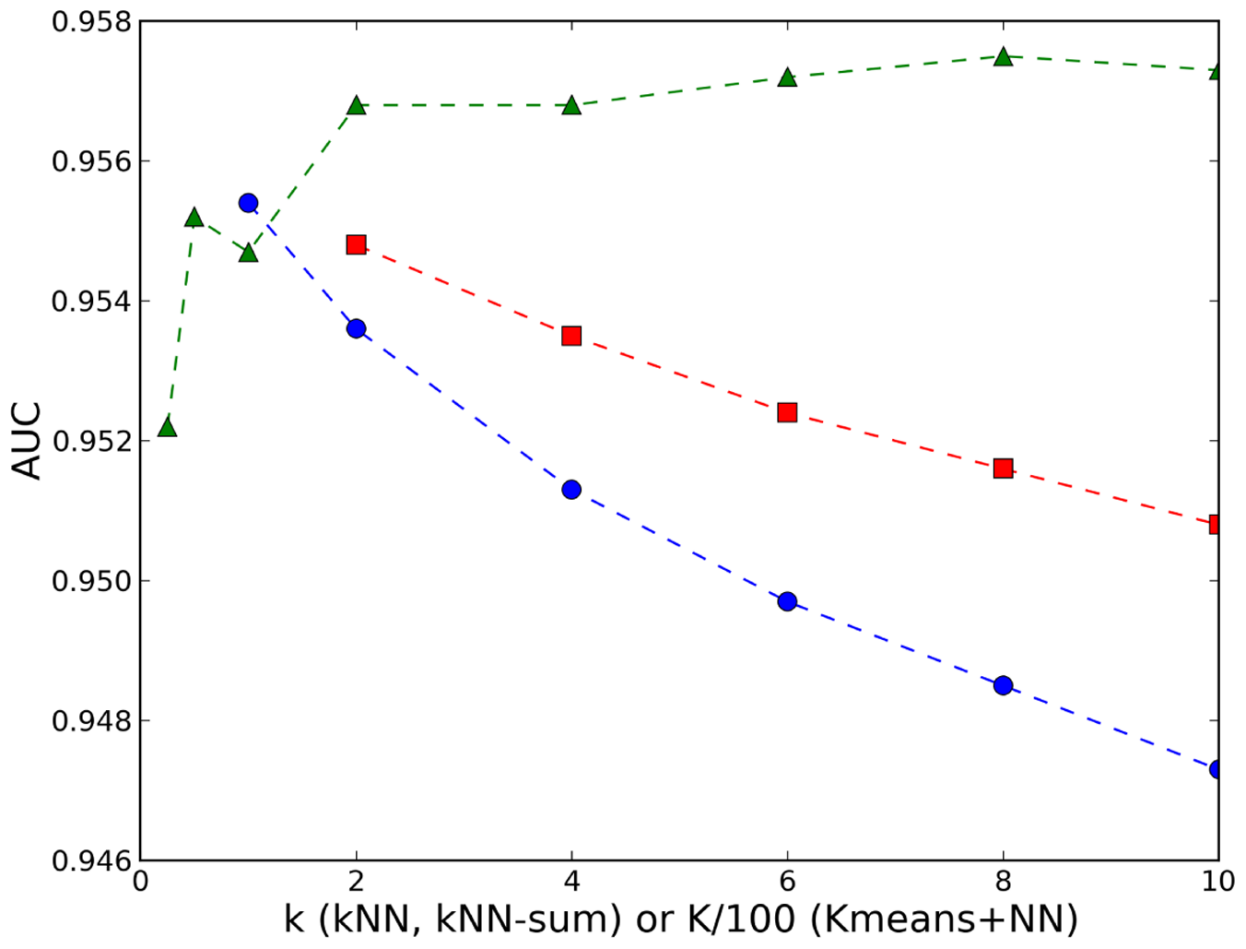
The AUC of kNN (blue points), kNN-sum (red squares) and K-means+NN (green triangles) for different values of k.

In figure 2 we compare the AUC of kNN, kNN-sum and K-means+NN for different values of k. Increasing k for kNN or summing the distances to the k nearest neighbours did not help to improve the results, while for K-means+NN the optimum was found at K = 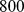, which roughly implies 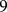 records per cluster.

In table 1 we present the results of the nearest neighbour-based methods (best k values) and those of the One-ClassSVM. In terms of AUC, One-Class SVM obtained the worst results (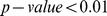 when comparing to each of the other three in a one-sided t-test). In terms of AUC, K-means+NN is the best. However, although the difference with respect to 1NN is statistically significant (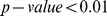), its value is about one order of magnitude smaller than the differences obtained in the comparison with SVM using the same data set (see below and table 2). Therefore, due to its simplicity for a smartphone application and to the ease of updating with new records, we picked 1NN for further analysis in our study.

**Table 1:** Comparison of novelty fall detectors.

Algorithm	AUC mean(std)	SE	SP
kNN k = 1	0.9554 (0.0052)	0.907	0.905
kNN-sum k = 2	0.9548 (0.0052)	0.913	0.901
K-means + 1NN (K = 800)	0.9575 (0.0056)	0.929	0.890
One-Class SVM	0.9439 (0.0060)	0.881	0.890

**Table 2:** Comparison of 1NN with SVM in terms of AUC (mean and std).

	SVM	1NN		
Conditions applied	AUC	AUC	Difference	p-value
Standard 10-fold CV	0.977 (0.010)	0.956 (0.011)	0.022 (0.006)	<0.01
Fall type-wise CV	0.976 (0.012)	0.956 (0.013)	0.020 (0.012)	<0.01
Phone sampling at 25 Hz	0.969 (0.008)	0.946 (0.010)	0.022 (0.007)	<0.01
Phone sampling at 16.7 Hz	0.961 (0.009)	0.937 (0.010)	0.024 (0.008)	<0.01
Phone in hand bag	0.899 (0.011)	0.951 (0.007)	−0.053 (0.007)	<0.01

Different conditions are considered in each row. The first row is the standard cross-validation (CV). In the second row the CV is done by leaving out each time a different type of fall for testing. In the remaining rows, the validation sets for CV are taken under varying conditions. 1NN is trained and tested with data obtained under the same conditions, while SVM is trained with data obtained under “standard” conditions (50 Hz, phone in pocket).

### Adapting 1NN and comparing with SVM

**Figure 3:**
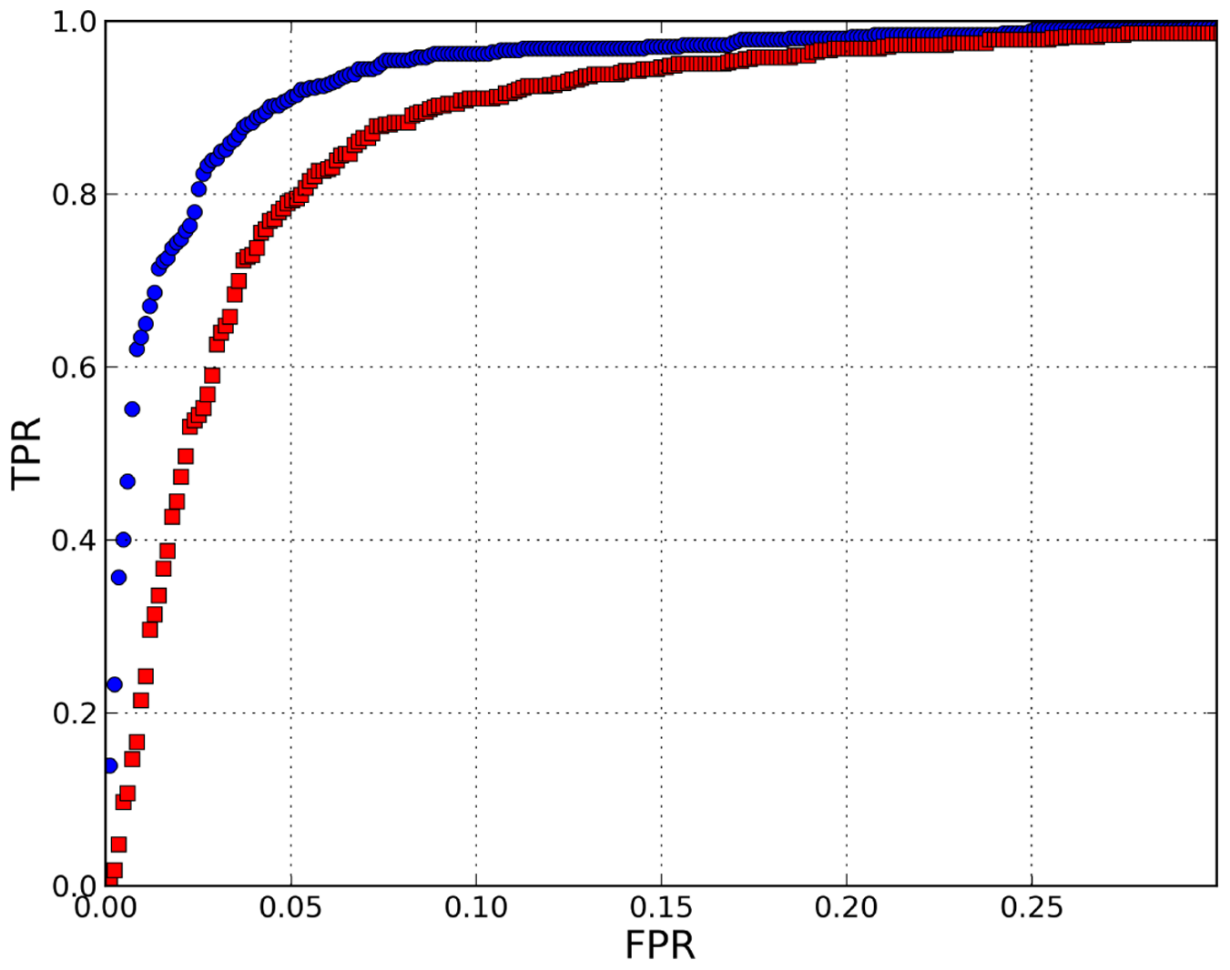
ROC curve for SVM (blue points) and 1NN (red squares).

We compared 1NN with a supervised method SVM, under different conditions as explained in section “Materials and Methods”. The results are shown in table 2 for AUC and in table 3 for specificity and sensitivity. The last column in table 2 is the p-value of a one-sided t-test applied to the difference in AUC. The first row is the result of the standard 10-fold cross-validation taking into account all the data. SVM clearly outperformed 1NN. This is also graphically shown in figure 3, in which the differences between the ROC curves can be appreciated. Until the true positive ratio (TPR) reaches a value of 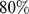, the ROC curve of 1NN is displaced with respect to that of SVM roughly up to a 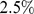 in the value of the x-axis, the false positive rate (FPR).

**Figure 4:**
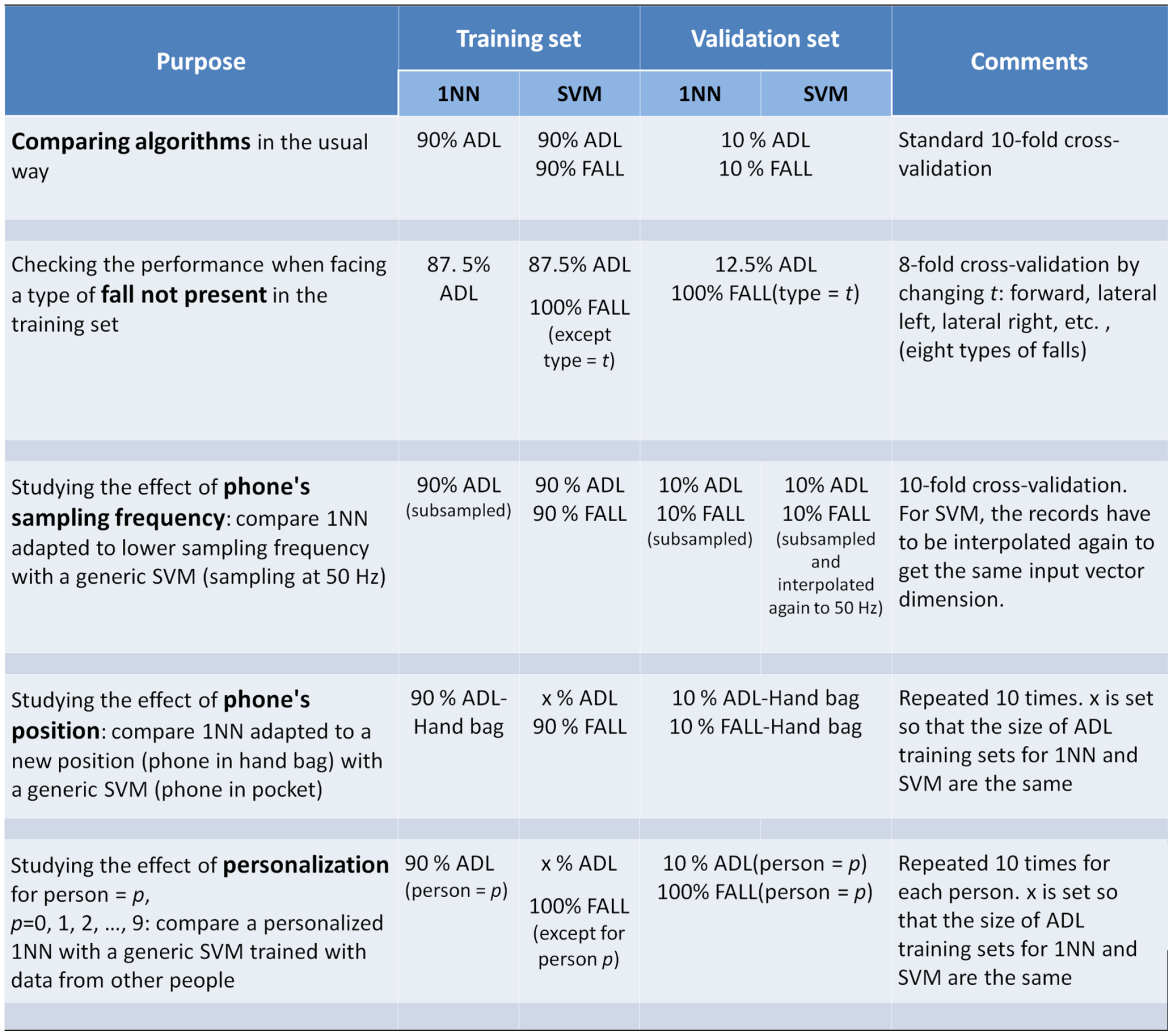
Schematic summary of cross-validation conditions. ADL and FALL represent the original data set (50 Hz, phone in pocket). Between parentheses, we add additional conditions. For instance, FALL(type = *t*) means the falls of a given type *t*. ADL-Hand bag and FALL-Hand bag are the data sets obtained while carrying the phone in a hand bag.

**Table 3:** Comparison of 1NN with SVM in terms of SE and SP.

	SVM			1NN		
Conditions applied	SE	SP	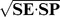	SE	SP	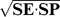
Standard 10-fold CV	0.954	0.924	0.939	0.910	0.903	0.907
Fall type-wise CV	0.953	0.926	0.939	0.904	0.915	0.909
Phone sampling at 25 Hz	0.930	0.918	0.924	0.891	0.901	0.896
Phone sampling at 16.7 Hz	0.893	0.919	0.906	0.895	0.880	0.887
Phone in hand bag	0.903	0.7912	0.845	0.910	0.893	0.902

Different conditions are considered in each row. The first row is the standard cross-validation (CV). In the second row the CV is done by leaving out each time a different type of fall for testing. In the remaining rows, the validation sets for CV are taken under varying conditions. 1NN is trained and tested with data obtained under the same conditions, while SVM is trained with data obtained under “standard” conditions (50 Hz, phone in pocket).

The results when the cross-validation was performed by fall type are presented in the second row of tables 2 and 3. Although there was a small decrease in performance, SVM still outperformed 1NN.

In the third and fourth rows of tables 2 and 3 we present the results of an analysis to study the effect of sampling frequency, again repeated 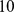 times for cross-validation. The performance of both SVM and 1NN deteriorated being 1NN still the worst.

In the last row of tables 2 and 3, we investigated the importance of the way the phone is carried. The adapted 1NN experienced a slight decrease in performance, while the generic SVM got clearly worse. In this case, 1NN outperformed SVM.

Finally, we studied the effect of personalization. In tables 4 and 5 the results for each volunteer are presented. P-values were obtained as explained above. Three cases are considered. In two of them the detector was generic, denoted as SVMG and 1NNG, while the last one was personalized, 1NNP. Concerning the comparison between SVMG and 1NNP, in eight out of ten people SVMG outperformed 1NNP, but for two people the personalized detector was a better option. In average, the difference between SVMG and 1NNP gets smaller when personalizing the detector, see the last row of table 4 and the first row of table 2. In table 5 we show the results in terms of SE and SP. In this table the geometric mean obtained using 1NNP is higher for four people. Concerning the effect of personalization on the novelty detector, the difference in AUC between 1NNP and 1NNG is positive for nine people and negative only for person 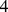, but being very small (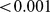) and without statistical significance (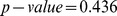).

**Table 4:** Comparison between generic SVM and 1NN detectors (SVMG, 1NNG) and a personalized 1NN detector (1NNP) in terms of AUC (mean and std).

	SVMG	1NNG	1NNP	SVMG-1NNP		1NNP-1NNG	
Person	AUC	AUC	AUC	Difference	p-value	Difference	p-value
Person 0	0.976 (0.007)	0.929 (0.017)	0.955 (0.013)	0.021 (0.009)	<0.01	0.026 (0.008)	<0.01
Person 1	0.986 (0.010)	0.974 (0.012)	0.979 (0.012)	0.007 (0.008)	0.014	0.005 (0.002)	<0.01
Person 2	0.941 (0.007)	0.941 (0.012)	0.950 (0.011)	−0.009 (0.012)	0.023	0.090 (0.004)	<0.01
Person 3	0.983 (0.012)	0.9410(0.014)	0.965 (0.009)	0.018 (0.011)	<0.01	0.024 (0.007)	<0.01
Person 4	0.963 (0.007)	0.954 (0.010)	0.953 (0.012)	0.009 (0.009)	<0.01	−0.000 (0.004)	0.436
Person 5	0.921 (0.022)	0.653 (0.053)	0.962 (0.013)	−0.040 (0.022)	<0.01	0.309 (0.046)	<0.01
Person 6	0.964 (0.014)	0.912 (0.024)	0.950 (0.020)	0.014 (0.013)	<0.01	0.038 (0.013)	<0.01
Person 7	0.971 (0.007)	0.952 (0.010)	0.965 (0.011)	0.007 (0.007)	<0.01	0.013 (0.005)	<0.01
Person 8	0.988 (0.007)	0.948 (0.022)	0.966 (0.019)	0.022 (0.018)	<0.01	0.019 (0.011)	<0.01
Person 9	0.988 (0.006)	0.945 (0.012)	0.977 (0.010)	0.011 (0.006)	<0.01	0.032 (0.009)	<0.01
Average	0.968	0.915	0.962	0.006		0.047	

For each person, the personalized 1NN is trained only with part of his or her own data, and tested with the remaining data. The generic SVM or 1NN in turn are trained with data from the remaining people but tested on the same validation set. This is repeated ten times for cross-validation.

**Table 5:** Comparison between generic SVM and 1NN detectors (SVMG, 1NNG) and a personalized 1NN detector (1NNP) in terms of SE and SP.

	SVMG			1NNG			1NNP		
Person	SE	SP	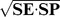	SE	SP	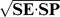	SE	SP	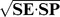
Person 0	0.908	0.946	0.927	0.827	0.871	0.849	0.867	0.925	0.895
Person 1	0.992	0.923	0.957	0.983	0.901	0.941	0.964	0.945	0.955
Person 2	0.861	0.942	0.900	0.892	0.894	0.893	0.932	0.894	0.913
Person 3	0.970	0.929	0.950	0.904	0.857	0.880	0.952	0.901	0.926
Person 4	0.877	0.939	0.907	0.944	0.878	0.911	0.952	0.876	0.913
Person 5	0.866	0.784	0.824	0.804	0.545	0.662	0.950	0.909	0.929
Person 6	0.961	0.859	0.909	0.898	0.831	0.864	0.950	0.859	0.903
Person 7	0.917	0.965	0.941	0.919	0.930	0.924	0.955	0.930	0.942
Person 8	0.961	0.953	0.957	0.900	0.884	0.892	0.953	0.907	0.930
Person 9	0.981	0.925	0.953	0.932	0.812	0.870	0.940	0.912	0.926
Average	0.929	0.917	0.922	0.900	0.840	0.869	0.941	0.906	0.923

For each person, the personalized 1NN is trained only with part of his or her own data, and tested with the remaining data. The generic SVM or 1NN in turn are trained with data from the remaining people but tested on the same validation set. This is repeated ten times for cross-validation.

## Discussion

In recent years there has been an increasing number of studies using smartphones to detect falls. Smartphones are suitable devices because they have built-in accelerometers, powerful processors and built-in communications protocols that allow alarms to be sent. Unlike previous studies using Machine Learning methods, in this paper we sought to test novelty detection methods to discriminate falls. In this way, we had a set of records, ADL, that represented the normal behaviour. Given a new record, we wanted to decide whether it belonged to the same distribution. If not, it should be classified as a potential fall and an alarm should be triggered. Being only based on true ADL, these methods seemed to be good candidates to adapt to varying conditions.

However, our guess was not confirmed. SVM outperformed 1NN when using all the records together. This was not surprising considering that SVM used all the information available, but the same result was still valid even if the cross-validation was made by type of fall. It seems that the degree of similarity between falls was high enough to allow SVM to classify all of them in the same group, regardless of their kind. Running SVM in a smartphone with lower sampling frequency than the frequency used for training was also better than an adapted 1NN. On the positive side of novelty detectors, our results show that SVM was very sensitive to the position of the phone. Previous studies always considered standardized positions. If the phone can be used as a phone and placed in different positions, an algorithm that can adapt itself to these situations can improve the results. For instance, if some people carried the phone in a hand bag, 1NN could learn this situation by recording new ADL and it would outperform SVM trained with data taken in the “assumed” position, the pocket.

After personalizing the detector, 1NN was able to beat SVM for some participants, but it is difficult to decide in advance who could be in that group. In average, the performances of both algorithms became closer and even the choice of the figure of merit (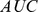 or 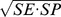) could change the selection of the algorithm for a given person. In contrast, personalizing 1NN clearly improved its results with respect to its generic version. According to these results, personalization is a valuable option which can increase the detector's performance. This aspect deserves further research in the future with a larger number of participants. For 1NN, customization could be easily achieved in the user's smartphone. However, for more complex algorithms, re-training the algorithm in the phone is likely to imply too much computation burden, but solutions considering the communication with an external server could be envisaged.

Concerning the relation with previous studies, most papers characterized the detector's performance using specificity and sensitivity. In the case of accelerometers attached to the body, even simple algorithms based on thresholds seemed to be almost perfect. For instance values as good as 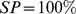 and 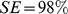 have been reported [7]. However, their performance under real-life conditions decreased dramatically as shown in [21]. In addition, accelerometers built into smartphones are unlikely to reach the range and accuracy of specialized devices. Our work can be compared more directly with studies based on smartphones. Using threshold based methods, Lee et al. [13] obtained 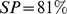 and 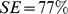, while Fang et al. [15] obtained 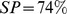 and 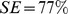. More sophisticated Machine Learning methods were used in [14], where accuracies of 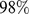 were reported. Other works achieved very good performance but relying on additional external sensors, like a magnetic accessory in [2], or another accelerometer in [16]. In our work, the average performance for 1NN in the base experiment was 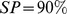 and 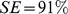, while SVM achieved 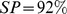 and 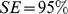, see table 3. Thus, we got better results than threshold-based methods, as expected due to their simplicity, but worse than those of [14]. This can be due to several reasons:

- The position of the smartphone was fixed on the back of the subject with a special belt in [14], while we just asked the volunteers to carry the phone in their pocket, thus being only loosely fixed. Besides, they could also use the phone to call.
- The features extracted were different. In [14] features such as moments, histograms or Fourier components were extracted. On the contrary we used the raw acceleration values.
- The time window was larger in [14], a time span of 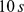. Although analyzing an extended period after the acceleration peak can help to reduce some false alarms, we feel that during falls simulated by volunteers the information included in the time before them is far from being realistic and it is highly conditioned by the researcher conducting the experiment. Falls themselves have been reported to be very short [24] and we took only 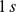 around the acceleration peak.
- Accelerometers might not have the same properties. For instance, in our experiments we did not sample any acceleration component higher than about 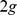.

The limitations of our study should also be acknowledged. We evaluated the algorithms using a restricted set of falls simulated by young and middle age people, all of them healthy subjects. Thus, they form a homogeneous group. It is still an open question if these records can be representative of older people's real falls since their movements are expected to be different from those of young or mature people [26], [27]. With regard to the information fed into the classifier, we have restricted this work to discriminate the acceleration shape during falls. Other features like the orientation change during the fall could help to reject many false alarms.

We must also mention some technical problems that we have faced when testing different mobile phones which could hinder the performance of a smartphone application. Smartphones were not originally intended for safety critical applications and special care is needed to ensure that programs run without interruption.

## Conclusions

This work was motivated by the possibility of using a smartphone for fall detection in real-world scenarios. We have explored a new type of approach based on novelty detection that allows an easy personalization of the detector because it is only trained with true ADL. We have compared it with a traditional SVM, which uses both falls and ADL for training. Even though a generic SVM has shown to perform better than an adapted NN in most of the situations that we have simulated using our public data base, the ultimate test should be carried out with real data from the elderly. This remains an opportunity for further research. We have shown that personalization boosts performance and this encourages us to test fall detectors with large groups of older people in real environments. Being different from laboratory set-ups, adaptability would be a key property to lower false alarms. Finally, novelty detection is a field with an intensive research where new algorithms and methods are being developed. Given that single algorithms are already very good in terms of error rate, our attention will turn towards the use of different features and combination of methods.
